# Involvement of Cisgender and Transgender Individuals in Studies on the Impact of Hormonal Therapy on COVID-19

**DOI:** 10.1089/apc.2020.0118

**Published:** 2020-09-03

**Authors:** Robert J. Wozniak, Douglas F. Nixon, Jez L. Marston

**Affiliations:** Division of Infectious Diseases, Department of Medicine, Weill Cornell Medicine, New York, New York, USA.

To the Editor:

Sex-based differences have shown to be an important aspect of COVID-19 susceptibility and disease course. A number of biological mechanisms have been proposed to explain these differences, including sex-based ACE2 receptor expression and DNA methylation,^1^ but lifestyle differences and comorbidities between cisgender men and women may also contribute. Because of these observations, new studies are exploring the safety and efficacy of sex hormones, specifically estrogen and progesterone, as COVID-19 treatments.^2^ This has some precedent from animal studies of the original SARS: in a study of SARS-CoV-1-infected mice, estrogen treatment led to decreased inflammatory reactions, virus titers, morbidity, and mortality.^3^ A new preprint also identified tamoxifen, an estrogen receptor antagonist, as a potential drug with activity against SARS-CoV-2 based on connectivity mapping and in silico binding studies.^4^ These findings are imperative to finding solutions to this evolving pandemic; however, the transgender community is being excluded from COVID-19 demographics data, research studies, and public health surveillance systems, ultimately limiting our understanding of how biological sex and the use of hormone modulators can impact the disease.

A potentially more efficient route to understanding hormone therapy in COVID-19 is to consider transgender and gender-nonconforming individuals who are currently utilizing hormone replacement therapy (HRT), which is used in the gender-affirming transition process. Male-to-female (MTF) HRT includes estrogens, progestogens, and antiandrogens, leading to the development of characteristically female attributes; female-to-male (FTM) HRT includes the administration of testosterone to suppress the production of estrogen and allow for the development of characteristically male attributes. Given the promising results of the animal model and estrogen receptor antagonist repurposing studies,^3,4^ it is possible that those undergoing MTF HRT have heightened protection from experiencing severe COVID-19, whereas those on FTM HRT may face an increased risk. However, this hypothesis may be complicated by other biological and behavioral factors. For example, the existence of two X chromosomes in cisgender women may offer a better immune response to viral infections than one X chromosome in transgender women due to its expression of TLR-7, a key component in pathogen recognition and the activation of innate immunity.^5^ Further, transgender individuals experience higher rates of cancer, tobacco use, and other chronic conditions,^6^ which have been attributed to the worse progression and outcome of COVID-19.^7^ Considering that a prominent feature of COVID-19 is systemic thrombosis and hypercoagulability,^8,9^ it should also be noted that HRT used in the gender-affirming transition process has been linked to a hypercoagulable state.^10^ However, the proposed or ongoing studies mainly involve estradiol patches, which have been associated with less of a venous thromboembolism risk than the full doses taken for feminization or postmenopausal HRT.^11^

Therefore, it is essential that transgender-identifying individuals are included in COVID-19 research initiatives and data collections, especially those involving the use of sex hormones. Owing to the current relationship between this vulnerable population and the health care community, there are extremely limited, if any, ongoing research efforts or developments regarding transgender health and COVID-19. Meeting the needs of transgender individuals and including them in future COVID-19 research developments not only upholds the ethical principle of treating every patient equally, but it offers an opportunity to advance our knowledge on COVID-19 while protecting people of all communities.

## Authors' Contributions

R.J.W., D.F.N., and J.L.M. conceived of the presented idea, developed the theory, and wrote the article. All authors discussed and contributed equally to the final article. As the corresponding author, R.J.W. had final responsibility for the decision to submit for publication.
